# Bacterial Quorum-Sensing Peptides as Immune Modulators Present in Systemic Circulation

**DOI:** 10.3390/biom13020296

**Published:** 2023-02-04

**Authors:** Anton De Spiegeleer, Amélie Descamps, Srinath Govindarajan, Julie Coudenys, Kevin Van der borght, Hannah Hirmz, Nele Van Den Noortgate, Dirk Elewaut, Bart De Spiegeleer, Evelien Wynendaele

**Affiliations:** 1 Translational Research in Immunosenescence, Gerontology and Geriatrics (TRIGG) Group, Ghent University, 9000 Ghent, Belgium; 2 Department of Geriatrics, Faculty of Medicine and Health Sciences, Ghent University Hospital, 9000 Ghent, Belgium; 3 Drug Quality and Registration (DruQuaR) Group, Faculty of Pharmaceutical Sciences, Ghent University, 9000 Ghent, Belgium; 4 VIB Inflammation Research Center, Unit for Molecular Immunology and Inflammation, Ghent University, 9000 Ghent, Belgium; 5 Department of Rheumatology, Faculty of Medicine and Health Sciences, Ghent University Hospital, 9000 Ghent, Belgium

**Keywords:** quorum-sensing peptides, microbiome, immune cells, J774, splenocytes, PBMC

## Abstract

Quorum-sensing peptides (QSPs) are bacterial peptides traditionally considered only as inter-bacterial communication molecules. Recently, their involvement in microbiome–host interactions influencing host diseases such as cancer and sarcopenia were explored. However, it is still unknown to what extent these peptides have the potential to modulate the immune system. In this proof-of-concept study, we screened 89 QSPs for their potential to induce IL-6 and TNFα in murine splenocytes and J774 macrophages. Confirmatory experiments on the positive screening-hits were conducted using murine splenocytes and human PBMCs of different ages. Finally, to investigate the biological relevance of immunomodulatory QSPs, we analysed plasma in a human cohort for the presence of the immunomodulatory QSP Q010. To do this, we used a newly developed UHPLC-MS/MS method. Our findings indicated that specific QSPs activate immune cells in vitro, with Q007, Q010, Q017 and Q212 being the top four screening hits. Q007 and Q010 were affirmed in subsequent confirmatory experiments using murine splenocytes and human PBMCs. Finally, Q010 was detected in human plasma, demonstrating for the first time the presence of an immunomodulatory QSP in human circulation. In conclusion, our data are the first evidence indicating the potential of biologically relevant quorum-sensing peptides to modulate the immune system.

## 1. Introduction

Healthy humans live in symbiosis with their bacterial inhabitants. In contrast, dysbiosis of the host microbiota has been implicated in different diseases [1]. Moreover, the (gut) microbiota have been suggested as a 10th hallmark of ageing, where the diseases associated with dysbiosis are considered signatures of accelerated ageing [2]. Inflammation, cognitive deterioration (up to dementia) and physical deterioration (up to sarcopenia) are examples of this dysbiosis-associated accelerated ageing. While most microbiota–host research has focused on lipids and small molecules such as vitamin B12, bacterial peptides are increasingly recognised as important microbiota–host mediators [3]. 

A group of bacterial peptides that recently came to attention in the context of microbiota–host interactions are the quorum-sensing peptides (QSPs). QSPs are peptides mainly produced by gram-positive bacteria, which are used for intra- and interspecies communication. They are produced as large pro-peptides and hydrolysed to the active QSP while leaving the bacterial cell through ATP-binding cassette transporters. Interaction with its bacterial neighbours occurs classically via membrane histidine kinase receptors coupled with transcription factors or directly by the intracellular binding of the QSP to transcription factors. These transcription factors initiate processes where the bacteria behave as multicellular organisms instead of individual bacteria, such as biofilm formation, competence and virulence [4]. Currently, more than 350 different QSPs are described [5]. 

Recently, it has been demonstrated that some of these QSPs can cross biological barriers such as the blood–brain barrier and the gut barrier [6,7,8]. Moreover, both in vitro as well as in vivo studies show biological effects of specific QSPs on the host. For example, EntF* (*E. faecium*) promotes colorectal cancer metastasis, iAM373 (*E. faecalis*) induces sarcopenia and PapRIV (*B. cereus*) activates BV-2 microglial cells [6,7,9,10]. Preliminary studies also suggest an effect of selected QSPs on immune cells. Sannomiya et al. showed that cAM373 and CPD1, both produced by *E. faecalis*, are potent chemotactic agents and inducers of lysosomal granule enzyme secretion in murine neutrophils in vitro [11], which was confirmed by Ember et al. in human neutrophils in vitro [12]. A recent study of Pundir et al. demonstrated mast cell activation after QSP binding to the G-protein-coupled receptor Mrgprb2. The seven identified mast cell activators were QSPs produced by *E. faecium* or *Streptococcus* species [13]. Although these studies point towards a QSP-immune interaction, no general immune screening of a broad array of QSPs has been conducted yet. In addition, no studies have been reported analysing the presence of immunomodulatory QSPs in human plasma. 

The aim of this proof-of-concept study was to systematically assess if QSPs have the potential to modulate the immune system. Therefore, we first screened a broad range of QSPs on immune activity in vitro, followed by confirmatory experiments on murine splenocytes and human PBMCs. Finally, using an optimised UHPLC-MS/MS method, we assessed the presence of a specific immunomodulatory QSP in human plasma. 

## 2. Materials and Methods

### 2.1. Study Design

A total of 89 QSPs were screened for their inflammatory activity on J774 cells and mouse splenocytes using IL-6 and TNFα ELISAs. Each QSP was independently studied at least four times (*n* = 4–15 biological replicates). The four strongest QSP hits, i.e., with the highest global z-score, were re-assessed at 3 different concentrations in confirmatory IL-6 experiments with splenocytes of different age. One confirmed QSP was evaluated for its presence in human plasma, and investigated for its association with specific cytokines in a subset of the human cohort. 

### 2.2. Quorum-Sensing Peptides

All peptides were purchased with a purity of minimally 95%. The suppliers were GL Biochem (Shanghai, China), LifeTein (Somerset, New Jersey, USA) and ChinaPeptides (Shanghai, China). The peptides were labelled by their Quorumpeps ID number [5], where the amino acid sequences can be found.

### 2.3. Animals

All experimental procedures were performed in accordance with institutional guidelines for animal studies and were approved by an ethics committee (ECD 19-17, approval 10/07/2019, University Hospital Ghent). C57Bl6/J WT mice were obtained from Janvier Laboratory (St-Berthevin, France). Mice were housed conventionally in a constant temperature (20–22 °C) and humidity (50–60%) animal room, with a 12 h–12 h light–dark cycle and food and water ad libitum. All mice were euthanised by cervical vertebra dislocation.

### 2.4. Splenocytes

Spleens were harvested (C57Bl6/j mice) and immediately pushed gently through a 70 µm strainer. After centrifugation (300× *g*, 22 °C, 8 min), red blood cells were lysed by incubation in an ammonium-chloride-potassium lysis buffer for 4 min. The cell suspensions were washed twice with cold phosphate-buffered saline (PBS) (300 g, 4 °C, 8 min) before being suspended in culture medium, composed of the following products: Dulbecco’s modified Eagle medium (DMEM; Thermo Fisher Scientific, Waltham, MA, USA) with 10% *v*/*v* fetal bovine serum (FBS; Thermo Fisher Scientific), 1% *v*/*v* L-glutamine (Thermo Fisher Scientific), 1% *v*/*v* penicillin/streptomycin (pen/strep; Thermo Fisher Scientific) and 50 mM ß-mercaptoethanol (Sigma-Aldrich, St. Louis, MI, USA). 

### 2.5. J774 Cells

J774 murine macrophages were grown in DMEM medium supplied with 10% *v*/*v* FBS and 1% *v/v* pen/strep solution. The cells were cultured at 37 °C and 5% CO₂. Approximately every 4 days, cells were split using a cell scraper.

### 2.6. Human PBMCs

PBMCs from 8 healthy adult individuals (*n* = 4 aged < 30 years; *n* = 4 aged > 60 years) were isolated by Ficoll density centrifugation (Histopaque, Sigma diagnostics, Delhi, India). The cells were grown in RPMI medium supplied with 10% *v*/*v* FBS and 1% *v*/*v* pen/strep solution. Our study followed the ethical principles of the Declaration of Helsinki and received approval from the ethical committee of the Ghent University Hospital (B670201734698, approval 19/01/2018, University Hospital Ghent). 

### 2.7. QSP Incubation

For the screening experiments, J774 cells and splenocytes were seeded at a concentration of 10,000 cells/well and 1,000,000 cells/well, respectively, in a 96-well plate. 24 h post-seeding, the cells were treated with 100 nM QSP for 24 h or 40 h. For the confirmatory experiments, splenocytes and human PBMCs were seeded at a concentration of 500,000 cells/well and 100,000 cells/well, respectively, in a 96-well plate. 24 h post-seeding, the cells were treated with different QSP concentrations (1 µM–10 µM–100 µM) or placebo for 24 h. The outer wells of the 96-well plates were filled with phosphate-buffered saline (PBS) to prevent evaporation effects. Supernatants of the experiments were stored at −80 °C until analysis. 

### 2.8. ELISA

IL-6 and TNFα levels were determined in the supernatants using a sandwich ELISA (Thermo Fisher Scientific, Waltham, Massachusetts, USA). Briefly, capture antibody, blocking solution, samples and standards, detection antibody, avidin-horseradish peroxidase and substrate solution were added sequentially, with washing steps in between. After stopping the reaction with H_2_SO_4_ solution, the absorbance was measured at 450 nm and 570 nm (correction wavelength) using a microplate reader. 

### 2.9. In Vitro Cell Medium and Human Plasma Stability

In vitro chemical and metabolic stability of Q007, Q010, Q017 and Q212 was determined in cell medium and human plasma, respectively, using previously described procedures [14]. In brief, 100 µg of peptide was incubated in Krebs–Henseleit buffer pH 7.4 with cell medium/plasma (500 µL) at 37 °C while shaking. At predetermined time intervals (i.e., 0, 6 and 24 h for cell medium; 0, 30 and 120 min for plasma), aliquots were immediately transferred into microtubes containing 1:1 volume of 1% (*v*/*v*) trifluoroacetic acid solution in water. The enzyme reaction was further stopped by heating the solution at 95 °C for 5 min. Next, the samples were centrifuged to precipitate the denatured proteins and the supernatant analysed using UPLC-PDA (Waters, Milford, MA, USA). Appropriate placebo solutions were similarly prepared. Assuming first-order kinetics, the rate constant k was obtained from ln(P_t_/P_t0_) = −kt, from which the half-life was determined as T_1/2_ = ln(2)/k.

### 2.10. Haemolysis Assay

In vitro haemolysis of Q007, Q010, Q017 and Q212 was determined using previously described procedures. Briefly, freshly obtained human blood was centrifuged and the precipitate washed using freshly prepared 150 mM NaCl solution. After the final centrifugation step, the precipitate was resuspended into 100 mM sodium phosphate buffer pH 7.4. A 1 to 10 RBC solution was incubated for 1 h at 37 °C with QSP at a concentration of 1 μM. The absorbance of the supernatant was measured at 405 nm and corrected for the blank absorbance. The percent haemolysis was then calculated using the following equation: [(Asample − Ablank)/(Apositive control − Ablank)] × 100, with 1% (*v*/*v*) Triton X-100 being the positive control. 

### 2.11. Caco-2 Experiments

Caco-2 permeability tests were performed as described by Wynendaele et al. [7], based on the protocol of Hubatsch et al. [14]. Briefly, Caco-2 cells were seeded on 12-well polycarbonate membrane filters (0.4 μm pore size, Corning, Teltow, Germany) and left for differentiation during 21–29 days. Peptide solutions (1 μM and 10 µM Q010) were added to the apical chamber, and aliquots were taken from the basolateral chamber immediately after (considered T_0_ min) as well as after 15 and 30 min of incubation. Samples were quantitatively analysed using UPLC-MS/MS. Four independent biological replicates were used (different days and well-plates), with each between 3 and 6 technical replicates (i.e., different wells on one plate/in one day). The global apparent permeability coefficient as the best central estimate and its standard deviation were calculated using the inverse variance weighing scheme.

### 2.12. Human Study 

The human cohort consisted of 64 adults (mean age, 62 years; IQR 59 years; female, 56%), from whom blood was collected, in addition to minimal demographic characteristics (age, sex and BMI). All participants were aged ≥ 18 years. Our study followed the ethical principles of the Declaration of Helsinki and received approval from the ethical committee of the Ghent University Hospital (B670201734698, approval 19/01/2018). Each participant signed a consent statement form after receiving written/verbal study information.

### 2.13. Sample Collection and Preservation

Human plasma was collected in K_2_EDTA-containing tubes (BD Vacutainer, Franklin Lakes, NJ, USA) and centrifuged at 1000× *g* for 10 min at 21 °C, within 10 min after withdrawal. The plasma supernatant was immediately transferred to 1.5 mL LoBind Eppendorf tubes on ice, and plasma sample preparation for Q010 analysis started within 20 min. 

### 2.14. Sample Preparation

Human plasma (900 µL) was mixed with 2.1 mL 2% HCl in acetonitrile/DMSO (97/3 ratio). After mixing for 30 sec and sonication for 30 s, samples were centrifuged for 1 min at 3000× *g* at 21 °C. The supernatant (2.5 mL) was subsequently transferred to a new tube and heated for 1 min at 100 °C. The solution was chilled on ice for 1 min and centrifuged for 1 min at 3000× *g* at 21 °C. Two millilitres of the supernatant was transferred to a new tube and immediately frozen on dry ice. Lyophilisation started within 4 h. The lyophilised sample (30 µL) was diluted in 970 µL of acetonitrile containing 0.1% formic acid. After mixing for 1 min and sonicating for 5 min, 900 µL supernatant was loaded on a HILIC amide SPE MonoSpin column previously conditioned with acetonitrile and equilibrated with the equilibration solution acetonitrile/DMSO (97/3 ratio) acidified by adding 0.1% formic acid. Samples were eluted using 120 µL of a mixture of 75/20/5 (*v*/*v*/*v*) water/acetonitrile/DMSO acidified with 0.1% formic acid. The eluent was finally transferred to a total recovery vial coated with anti-adsorption diluent, prepared as previously described [15].

### 2.15. QSP Detection

Q010 was detected and quantified on a Waters Acquity^®^ UPLC H-class system, connected to a Waters Xevo™ TQ-S triple quadrupole mass spectrometer with electrospray ionisation (ESI, operated in positive ionisation mode). Autosampler tray and column oven were kept at 10 ± 5 °C and 60 ± 5 °C, respectively. Chromatographic separation was achieved on a Waters Acquity^®^ UPLC BEH Peptide C_18_ column (300 Å, 1.7 µm, 2.1 mm × 100 mm), protected with guard column. The mobile phases consisted of 93/2/5 water/acetonitrile/DMSO (*v*/*v*/*v*) containing 0.1% formic acid (mobile phase A) and 2/93/5 water/acetonitrile/DMSO (*v*/*v*/*v*) containing 0.1% formic acid (mobile phase B), and the flow rate was set to 0.5 mL/min. A 10 µL aliquot from each sample was injected. The gradient started with 80% of mobile phase A for 30 s, followed by a linear gradient to 55% of mobile phase A for 5 min. Gradient was then changed to 14% mobile phase A at 5.5 min, followed by a 30 s equilibration, followed by a return to the starting conditions. Q010 showed retention at 5.10–5.75 min. An optimised capillary voltage of 3.00 kV, a cone voltage of 20.00 V and a source offset of 50.0 V were used in the ESI. Acquisition was done in multiple reaction monitoring (MRM) mode. The selected precursor ion was *m*/*z* 838.40 with two selected product ions: *m*/*z* 539.25 (21 eV, y_4_ fragment) as quantifier and *m*/*z* 673.27 (21 eV, b_6_ fragment) as qualifier.

A sample was considered positive for the presence of Q010 when the following three criteria were met: a signal appeared at the correct retention time for both quantifier and qualifier, similar to the pre-spiked reference sample, both quantifier and qualifier had a signal-to-noise ratio of minimally three (limit of detection) and the signal of the quantifier was higher than the signal of the qualifier.

### 2.16. Cytokine Multiplex

Bead-based multiplex LEGENDplex^TM^ analysis (LEGENDplex^TM^ Human Proinflammatory Cytokine Panel 13-plex; Biolegend, San Diego, California, USA) was used according to the manufacturer’s instructions. Reactions were performed in duplicates. Analysis was performed with the Cytoflex flow cytometer (Beckman Coulter, Krefeld, Germany). Data were analysed via LEGENDplex V8.0 software (Biolegend) and specified as pg/mL. 

### 2.17. BLAST

Amino acid sequence Q010 was blasted against the NCBI non-redundant (nr) database by Basic Local Alignment Search Tool (BLASTp). The organism was limited to human. Because of the short sequences being blasted, the E-values were not restricted. 

### 2.18. Data Analysis

All data analyses and graphs in this study were performed with RStudio Version 1.4.1717 and Illustrator Version 2021. In the screening, robust z-scores were calculated for each experiment as following: $0.675*\frac{\left(x_{i}-median\right)}{MAD}$, with MAD being the median absolute deviation. A global inflammatory z-score was calculated for each QSP using Stouffer’s method, i.e., global inflammatory z-score = $\overset{n}{\underset{i=0}{\sum }}z_{i}/\surd n$ [16]. In the confirmatory experiments, two-sided *t*-tests were conducted, where * *p* ≤ 0.05, ** *p* ≤ 0.01 and *** *p* ≤ 0.001. The association of cytokine concentration with the presence of Q010 in human plasma was assessed using linear regression models, after propensity score-overlap weighting for age, sex and BMI. This statistical procedure has been shown to mimic important attributes of randomised clinical trials, those being a clinically relevant target population, covariate balance and precision [17,18]. Data were presented as mean ± standard error of the mean (s.e.m.) unless otherwise indicated.

## 3. Results

### 3.1. In Vitro Screen of Inflammatory QSP Hits

**Figure 1:**
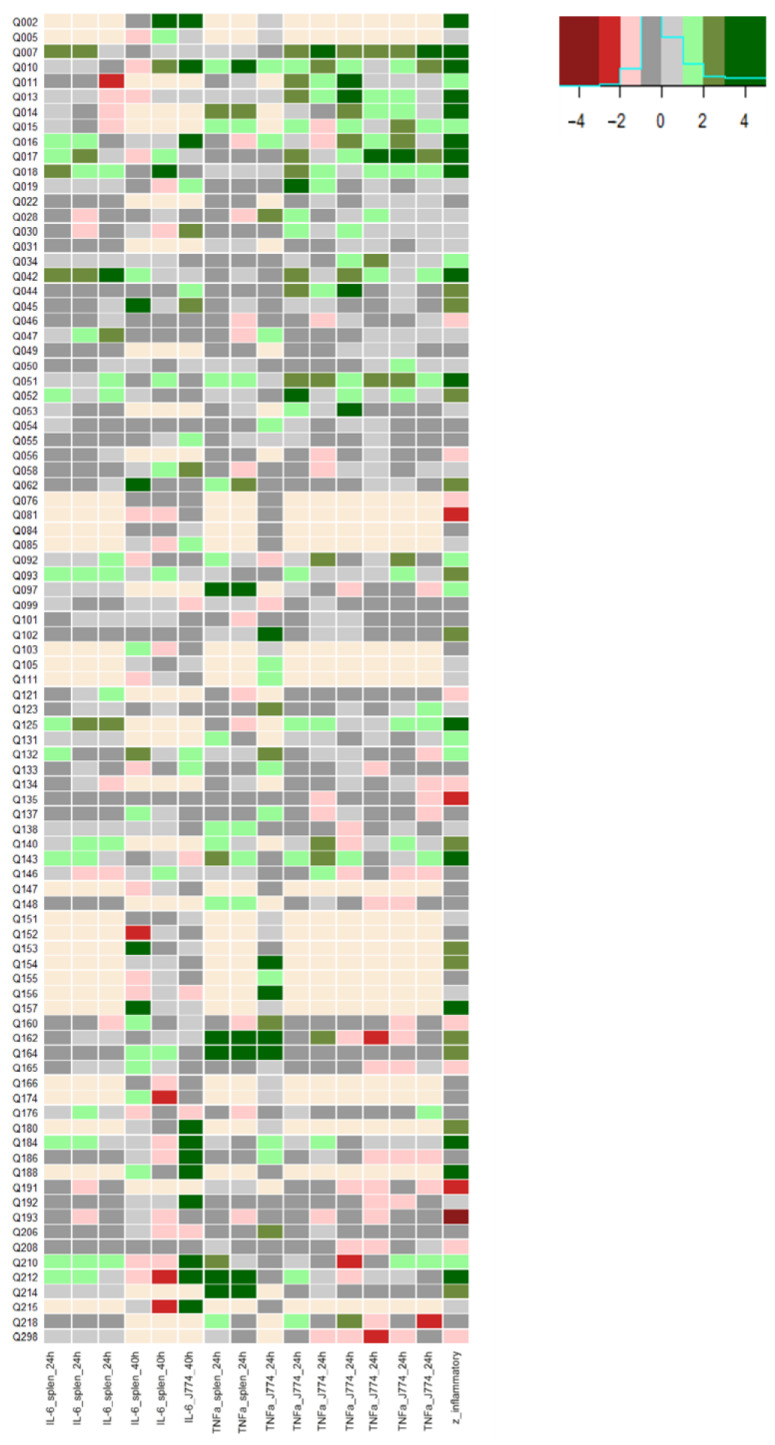
Heat plot of inflammatory screening on splenocytes and J774 cells. The *y*-axis represents all QSPs, while the *x*-axis represents the different experiments and the global inflammatory z-score (rightmost column). Each experiment is named based on its measured cytokine (IL-6 or TNFα), used cell type (spleen or J774) and incubation time (24 or 40 h). QSP concentration is 100 nM for all experiments. The colours are based on the z-score for each QSP in a specific experiment or overall for the column “z_inflammatory”. Missing data are indicated in faint yellow.

We screened 89 QSPs for their ability to modulate IL-6 and TNFα secretion in murine splenocytes—a mix from immune mononuclear cells derived from the spleen including T-lymphocytes, B-lymphocytes, NK-cells and NKT-cells—and murine macrophages (J774) (Figure 1). A combined inflammatory z-score was calculated based on 4–15 independent experiments (each column in Figure 1 is one independent experiment), performed over a wide time span to increase the reproducibility and confidence in the conclusions. The peptides Q007, Q010, Q212 and Q017 showed the highest inflammatory z-score, with values 6.9, 6.0, 4.8 and 4.6, respectively, obtained. The characteristics of these four peptides are shown in Table 1. 

### 3.2. The Inflammatory Activity of Selected QSP Hits Was Confirmed and Dose-Dependent 

**Figure 2:**
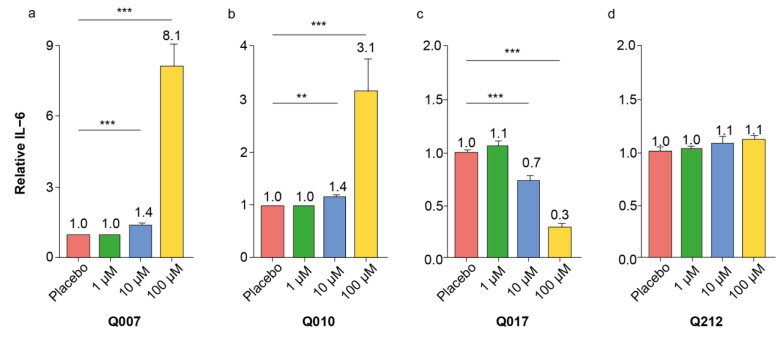
Results of IL-6 ELISA after 24 h incubation of splenocytes with different concentrations of QSP. (**a**) Q007; (**b**) Q010; (**c**) Q017 and (**d**) Q212. Bars and numbers on top represent means, with error bars representing s.e.m. Asterisks indicate the following criteria of statistical significance: ** *p* ≤ 0.01 *** *p* ≤ 0.001 (two-sided t-test between placebo and specific QSP concentration).

The four QSP with the highest overall inflammatory z-scores (Q007, Q010, Q017 and Q212) from the different screening experiments were re-assessed at different concentrations (1 µM, 10 µM and 100 µM) on splenocytes of different ages. Figure 2 shows the pooled results, while an exploratory subgroup analysis per age is provided in Supplementary Figure S1. The IL-6 pro-inflammatory effects of Q007 and Q010 could be confirmed, which was not the case for Q017 and Q212; Q017 even showed a decreasing apparent IL-6 response with increasing QSP concentrations (Figure 2). The IL-6-increasing effect of Q007 was mainly observed in middle-aged splenocytes (12 months old), while Q010 showed the strongest effect in young splenocytes (Supplementary Figure S1). Moreover, we performed experiments with scrambled quorum-sensing peptides Q007 (peptide sequence NFSPTWY) and Q010 (peptide sequence EAFFDLP), demonstrating QSP sequence specificity of the IL-6 effects (Supplementary Figure S2).

**Figure 3:**
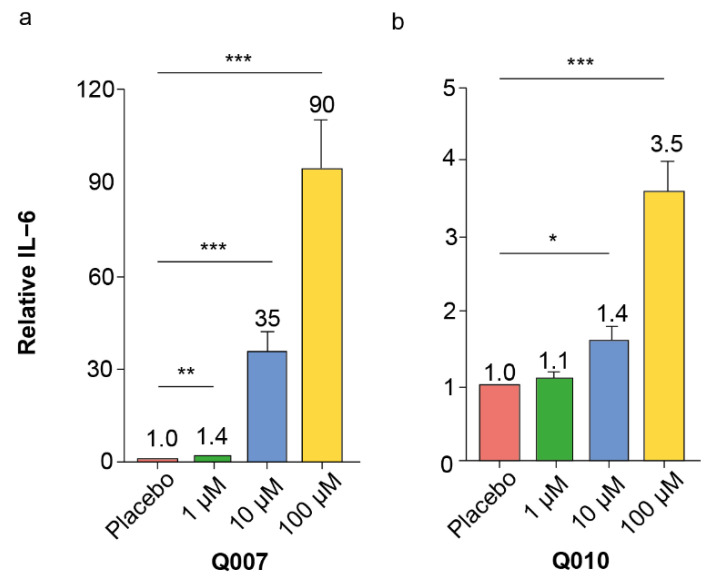
Results of IL-6 ELISA after 24 h incubation of human PBMCs with different concentrations QSP. (**a**) Q007; (**b**) Q010. Bars and numbers on top represent means, with error bars representing s.e.m. Asterisks indicate the following criteria of statistical significance: * *p* ≤ 0.05 ** *p* ≤ 0.01 *** *p* ≤ 0.001 (two-sided t-test between placebo and specific QSP concentration).

To translate the mice splenocyte immunomodulatory effects of Q007 and Q010 to the human setting, QSP experiments with human peripheral blood mononuclear cells (PBMCs) were conducted. After 24 h of Q007 or Q010 incubation, human PBMCs increased IL-6 secretion in a QSP dose-dependent way (Figure 3). Exploratory subgroup analysis per age showed a superior IL-6 response in young adults compared to older adults (Supplementary Figures S3 and S4).

When investigating the physiological relevance of peptides, metabolic stability in bio-fluids and general toxicity are important variables. Moreover, this kinetic and toxicity information is indispensable for future in vivo studies. The in vitro stability of Q007, Q010, Q017 and Q0212 was investigated in cell medium and human plasma. The half-life values of the quorum-sensing peptides in cell medium ranged from relatively short (i.e., less than 6 h) to very high (i.e., more than 160 h). The same was true for the human plasma stability, with T_1/2_ values ranging from less than 15 min to over 9 h (Table 2). As an initial toxicity assessment, haemolysis of human red blood cells was evaluated. Haemolysis was less than 1% for all four QSPs after a 1 h incubation period, indicating no general toxicity (data not shown). 

Taken together, these in vitro data confirm the inflammatory effects of specific QSP, with a dose-dependent effect. Moreover, plasma stability is high for specific immunomodulatory QSPs, in particular Q010.

### 3.3. Q010 (PapR7I) Was Detected in Human Plasma Using UPLC-MS/MS

**Figure 4:**
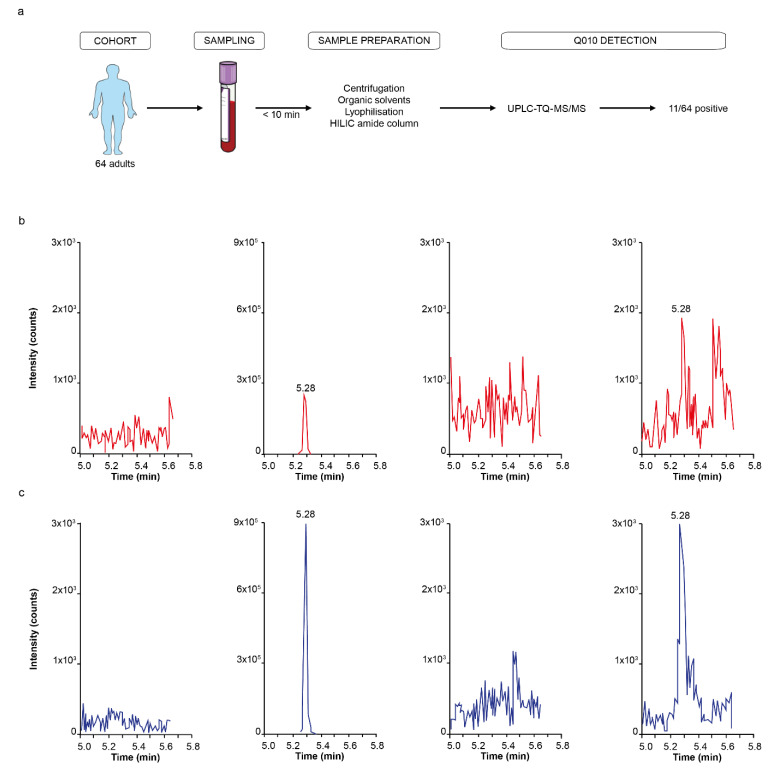
In vivo presence of Q010 quorum-sensing peptide in human plasma. (**a**) Flow chart of human in vivo data acquisition, from sampling of 64 adult plasma samples to LC-MS detection. (**b**) UPLC-TQ-MS/MS parent (*m*/*z* 838.4)- > daughter (*m*/*z* 673.3) qualifier chromatograms of a blank, pre-spiked (1 nM), negative and positive human plasma sample for Q010. (**c**) UPLC-TQ-MS/MS parent (*m*/*z* 838.4)- > daughter (*m*/*z* 539.3) quantifier chromatograms of a blank, pre-spiked (1 nM), negative and positive human plasma sample for Q010.

To investigate the presence of the metabolic stable Q010 (PapR7I) in vivo, EDTA plasma samples from 64 adults were collected and analysed using Reversed-Phase Ultra-high Performance Liquid Chromatography Triple Quadrupole Mass Spectrometry (RP-UPLC-TQ-MS). The characteristics of this MS/MS method, including process efficiency, recovery and matrix effect, are presented in Supplementary Table S1. Using this method, 11 out of 64 adults (17%) were considered positive for PapR7I. Explorative subgroup analysis in a young and old subgroup showed that 3 out of 25 young adults (12%) and 8 out of 39 older people (21%) were PapR7I-positive, a difference that was statistically not significant (Figure 4). Measured concentrations were between 0.3 and 2.5 pM. A human BLASTP search for PapR7I did not show any human sequence identical to PapR7I, indicative of not being a human endogenous cryptide and confirming a bacterial origin of the peptide observed in the plasma. Furthermore, Caco-2 in vitro experiments confirmed the gut permeability characteristics of Q010 with a global apical–basal apparent permeability coefficient (Papp) of 1.29 × 10^−8^ cm/s (s.e.m. = 0.67 × 10^−8^ cm/s; *n* = 4). The plasma of the older subgroup was also exploratively assessed for cytokine concentrations with a multiplex assay. Although an increase in IL-6 was observed with detectable Q010 (mean increase in IL-6 of 1.38 pg/mL; 95% CI −4.28 to 7.05), this was statistically not significant. The only statistically significant effect observed was a decrease in IFN-γ with detectable Q010 (−2.21 pg/mL; 95% CI −3.95 to −0.46) (Supplementary Table S2).

## 4. Discussion

Bacterial Quorum-Sensing Peptides (QSPs) are a group of bio-active peptides which are currently being explored for their possible interactions with mammalian host cells. In this proof-of-concept study, we screened 89 QSPs for their inflammatory potential and showed for the first time the presence of an immunomodulatory QSP in human plasma. 

The QSP selection for screening was based on the chemical space of the currently described QSPs, i.e., a chemically diverse set of QSPs was chosen [19]. Different IL-6 and TNFα experiments were conducted on both splenocytes and J774 macrophages. Splenocytes were chosen for this proof-of-concept study because they consist of a physiological mixture of immune cell populations. Given that splenocytes are largely immune cells of lymphoid origin, we added myeloid J774 cell experiments to the screening [20]. Both IL-6 and TNFα are pro-inflammatory cytokines produced by a broad range of immune cells, and with pleiotropic effects [21]. The short and controlled production of IL-6 and TNFα contributes to host defence against acute environmental stress. However, dysregulated, continuous IL-6 and TNFα production is involved in various health diseases, such as autoimmune diseases, cancers and muscle wasting [22,23]. Thus, targeting IL-6 and/or TNFα offers approaches to the diagnosis and treatment of different primary diseases (e.g., rheumatoid arthritis and sarcopenia) and health-compromising secondary conditions (e.g., cancer cachexia and post-infectious acute respiratory distress syndrome, ARDS). For this proof-of-concept investigation, we searched in our screening study for the QSPs at a 100 nM concentration with the clearest inflammatory response without making a distinction between IL-6 or TNFα response nor between splenocyte or J774 effect. Using 100 nM concentration is common in QSP initial screenings [24,25]. This concentration is at the lower spectrum of in vitro bio-active concentrations, but was justified due to quantities required at higher concentrations, making the initial screening too costly otherwise. Using this concentration, we might have missed some positive hits, but this is accepted due to the proof-of-concept design of this research. Moreover, an overall inflammatory score was applied covering all screening experimental results, minimising the probability of type II errors. Indeed, as expected and observed in Figure 1, some screening experiments did not show the effect of a specific QSP while others did, which was ‘averaged’ in the overall inflammatory score. From the screening experiments, Q007, Q010, Q017 and Q212 were identified as the QSPs with the highest overall inflammatory score. 

It is well-known and accepted that screening experiments have a non-negligible risk of false positive hits, which should be eliminated in subsequent confirmatory experiments [26]. Our confirmatory dose–response experiments with splenocytes of different ages confirmed the pro-inflammatory effects of Q007 and Q010, while those of Q017 and Q212 could not be confirmed. Moreover, the pro-inflammatory effects of Q007 and Q010 were also observed in human PBMCs, strengthening the potential human relevance of our findings. 

Q007 or RNAIII Inhibiting Peptide (RIP) is produced by different *Staphylococcus* species, commensal bacteria abundantly present on human skin and able to elicit disease in certain conditions [27]. Within bacteria, RIP inhibits the formation of RNAIII, a polycystronic transcript with four potential open reading frames, with one of them encoding haemolysin. RNAIII is also a regulatory RNA molecule upregulating the expression of other different toxins. Hence, RIP analogues are investigated for their potential to inhibit *S. aureus* infections [28]. Although the sequence YSPWTNF is often used to represent the native RIP, there is some ongoing discussion about the exact sequence of the native RIP molecule [29]. It is probable that different variants of RIP exist dependent on the species and strains. 

A strong inflammatory IL-6 response was observed after Q007 incubation in both murine splenocytes and human PBMCs. This effect was most pronounced in middle-aged mice (12 months old) and young adults (20–30 years old). This young adult age category would correspond to mice between 3 months and 6 months old [30]. These explorative data suggest an age-dependent effect for the inflammatory effect of Q007; however, in-depth studies with more head-to-head age categories in rodents and humans are needed to confirm and elucidate this. Sequence and cell target specificity are characteristics of most bio-active peptides [31]. Accordingly, a loss of activity was observed in murine splenocytes after scrambling the amino acid sequence of Q007. Cell target specificity needs to be further elucidated, as we previously showed no IL-6 response of Q007 in microglial cells in contrast to a potential IL-6 response in murine muscle cells [9,24]. To be biologically relevant in the microbiome–host immune interaction, Q007 needs to reach host immune cells. Assuming its production by the *Staphylococcus* species residing in human skin, in vitro and/or in vivo skin permeability experiments or proof of its presence in systemic circulation are needed. While no quorum-sensing peptide has yet been demonstrated to cross the skin barrier and enter systemic circulation, skin permeability data have already been described for other microbial peptides [32]. A ‘weakness’ of peptides is their relative instability [31]. Once Q007 has reached blood circulation, it probably remains sufficiently stable (in vitro T_1/2_ of 2 h), suggesting that this pharmacokinetic property is not the critical parameter for its biological relevance. In addition, Q007 did not induce haemolysis, i.e., no main general toxic effect was demonstrated. 

Q010, also called PapR7I, is a heptapeptide (ADLPFEF) originating from *Bacillus cereus* and *Bacillus thuringiensis* strains [33]. The *Bacillus cereus* group is widespread in soil and food. It is considered an opportunistic human pathogen because it can trigger food-borne gastroenteritis. However, *B. cereus* strains have also been demonstrated in symbiosis with human hosts, i.e., gastro-intestinal presence without provoking illness [34]. The heptapeptide is cleaved from the 48 amino acid-long PapR pro-peptide, and exerts its bacterial communication activity through direct binding to the intracellular transcription factor PlcR. PlcR regulates the expression of different virulence genes such as enterotoxins, cytotoxins and haemolysins [35]. 

This QSP showed a clear IL-6 response in our validation experiments, more outspoken in the young splenocytes and PBMCs. Although we are aware that differences exist between mouse and human immune cells, as well as between mice strains and sexes, some general immunosenescence principles seem to be shared. For example, there is a decrease in naïve (CD44-) T-cells and increase in memory (CD44+) T-cells with ageing, observed both in humans and mice. Additionally, the inversion of the CD4/CD8 ratio seems to be a general ageing phenotype. Concerning the innate immune system, the increased inflammatory activation modus is a recurring item in both mice and humans [36,37]. Without elucidating the mechanisms, our results roughly suggest a stronger pro-inflammatory effect of Q010 on the young immune system compared to the aged immune system, shared between mice and humans. Similar to Q007, Q010 showed sequence specificity, i.e., a loss of activity was observed when the amino acids were scrambled. Furthermore, we previously demonstrated that Q010 did not induce an IL-6 response in microglial cells or murine muscle cells, indicating cell specificity [9,24]. These data, in addition to the high plasma stability of Q010 (in vitro T_1/2_ of 9 h) and the lack of haemolysis-inducing effects, make Q010 a good potential candidate to be a biologically relevant immunomodulatory QSP. 

Accordingly, using a validated UHPLC-MS/MS approach, we were able to detect Q010 in the pM range in human plasma samples. Although the measured human plasma Q010 concentrations are low compared to the concentrations used in vitro, the total QSP exposure is in a similar range. Total exposure time is expected to be much higher in vivo, where a lifetime exposure of 70 years can be anticipated as recommended in relevant regulatory guidance [38], compared to the 24 h incubation time in vitro, resulting in similar exposure (i.e., concentration x time). For example, a plasma concentration of 2.5 pM for 70 years equals an exposure of 1.53 µM.h, similar to an in vitro exposure of 2.4 µM.h when incubating cells for 24 h with 100 nM Q010. Moreover, higher Q010 plasma concentrations are likely when investigating a larger cohort and/or specific populations; in this proof-of-concept, only 64 community-living people were investigated. Finally, it is also acknowledged that we have used a reductionistic approach in this proof-of-concept, i.e., looking only at one QSP (Q010) for its human presence, while we might expect other (even yet unknown) QSPs to additionally act on the immune system and be present in the systemic circulation. In line with the findings in human plasma, we demonstrated Q010 gut permeability in vitro using a human Caco-2 monolayer. The Papp of Q010 (1.29 × 10^−8^ cm/s) was within the range obtained for other QSPs: 1.4 × 10^−7^ cm/s for iAM373 [10] to 1.4 × 10^−9^ cm/s for PapRIV [6]. 

Exploratively, we also measured some cytokines in the plasma of an older subgroup, showing an increasing trend in IL-6 and a significant decrease in IFN-γ in the people with detectable Q010. This inversely proportional concentration of inflammatory cytokines IL-6 and IFN-γ in vivo is not uncommon. For example, an increased plasma IL-6 and decreased IFN-γ has been observed in ageing mice as well as humans with muscle wasting diseases such as cancer cachexia and sarcopenia [37,39,40]. In our ex vivo experimental splenocyte and human PBMC set-ups, IFN-γ concentrations in the supernatant were all below the limit of detection (data not shown). Hence, no conclusions can currently be made regarding whether Q010 has the potential to directly stimulate IFN-γ production in immune cells.

Our research has some limitations. First, the screened QSPs in this study are only a fraction of the currently described QSPs; moreover, novel QSPs are continuously being discovered. For example, a currently unexplored QSP group but with interesting structural features are the lipo-QSPs, such as Q023 and Q064 in the Quorumpeps Database [5]. Moreover, variations in only one amino acid of a QSP can be sufficient to shift the QSP’s biological activity towards the host [7]. This study is, thus, to be considered a proof-of-concept that QSPs can contribute to the microbiome-induced effects on the immune system and, hence, stimulate research in other existing or not yet discovered QSPs. Furthermore, no in vivo experiments were conducted to confirm causal QSP effects on the immune system, nor were experiments conducted elucidating the mechanisms of immune activation. In this regard, the data presented above represent a starting point for future in vivo and mechanistic studies. Despite these limitations, this study suggests for the first time that QSPs are potential modulators of the immune system present in the systemic circulation, bringing new research perspectives with theranostic opportunities. 

## Figures and Tables

**Table 1:** Four QSPs with the highest inflammatory score.

QSP	Trivial Name	Bacteria	Sequence
Q007	RNAIII Inhibiting Peptide (RIP)	*Staphylococcus aureus*, *Staphylococcus epidermidis*	YSPWTNF(-NH_2_)
Q010	PapR7I	*Bacillus cereus*, *Bacillus thuringiensis*	ADLPFEF
Q017	iPD1	*Enterococcus faecalis*	ALILTLVS
Q212	PapR5III	*Bacillus cereus*	VPFEF

**Table 2:** Plasma and cell medium half-life values of four QSPs.

QSP ID	Plasma Half-Life (h)	Medium Half-Life (h)
Q007	2.2	5.6
Q010	9.2	>160
Q017	2.5	29.8
Q212	0.2	26.0

## Data Availability

Raw data used in this study are fully available on request from the corresponding author.

## Supplementary Materials

The following supporting information can be downloaded at: https://www.mdpi.com/article/10.3390/biom13020296/s1, Figure S1: QSP splenocyte IL-6 results by age; Figure S2: Scrambled QSP IL-6 results; Figure S3: Q007 human PBMC IL-6 results by age; Figure S4: Q010 human PBMC IL-6 results by age; Table S1: MS/MS characteristics Q010 plasma detection; Table S2: Plasma cytokines associated with Q010 presence.
