# Supplementary material for: Bacterial Quorum-Sensing Peptides as Immune Modulators Present in Systemic Circulation
Source: Biomolecules. 2023 Feb 4;13(2):296. doi: 10.3390/biom13020296 (PMC9953703; doi:10.3390/biom13020296)
Supplement: Supplementary file 1 [file biomolecules-13-00296-s001.zip › biomolecules-2144944-supplementary.pdf]

Supplementary Information

Supplementary Figure S1

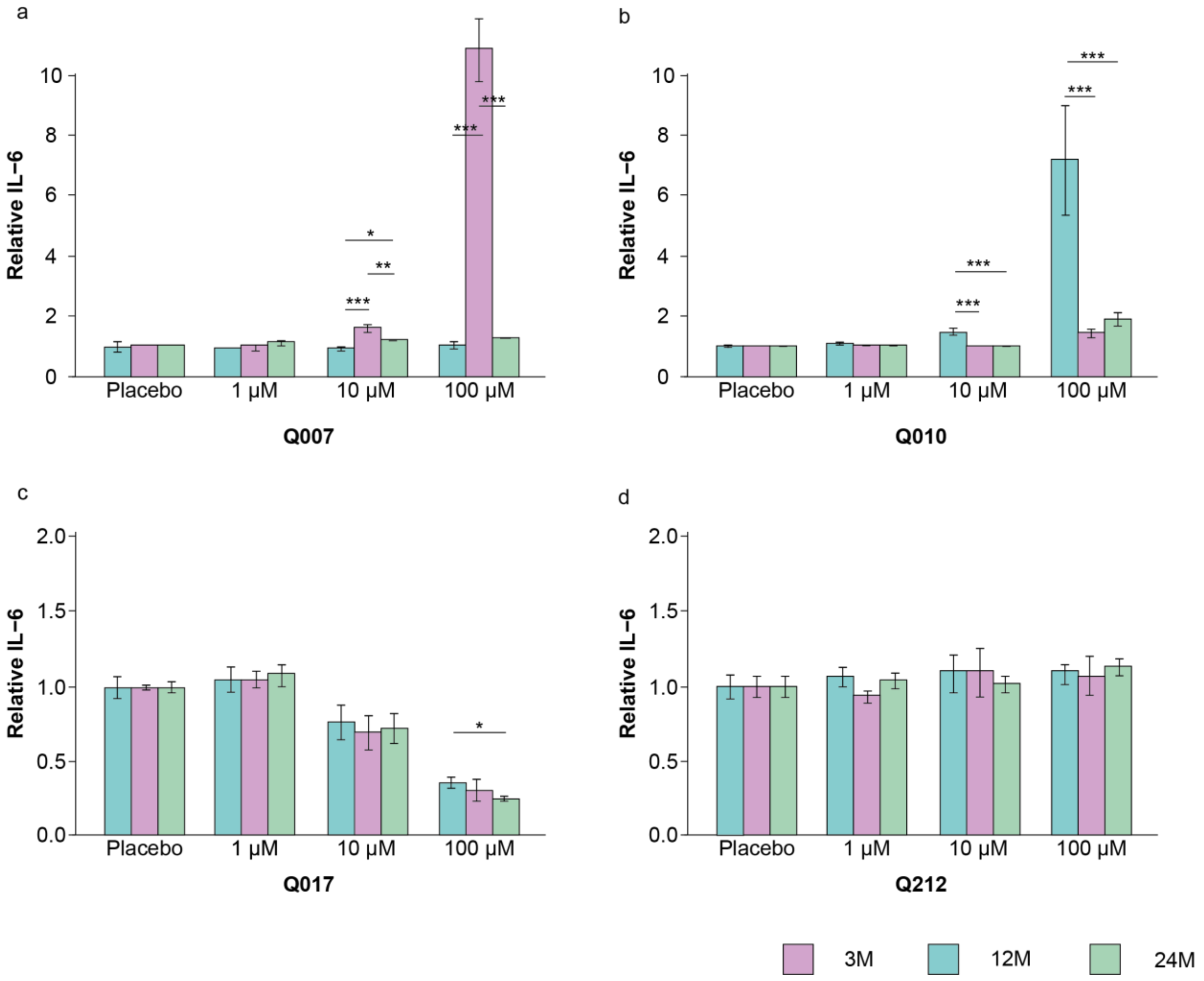

Supplementary Figure S1. Age-dependent results of IL-6 ELISA after 24h incubation of splenocytes with different concentrations QSPs. a) Q007; b) Q010; c) Q017 and d) Q212. Bars and numbers on top represent means, with error bars representing s.e.m. Asterisks indicate the following criteria of statistical significance: *p≤0.05 **p≤0.01 ***p≤0.001 (Two-sided t-test between different ages within the same QSP concentration).

Supplementary Figure S2

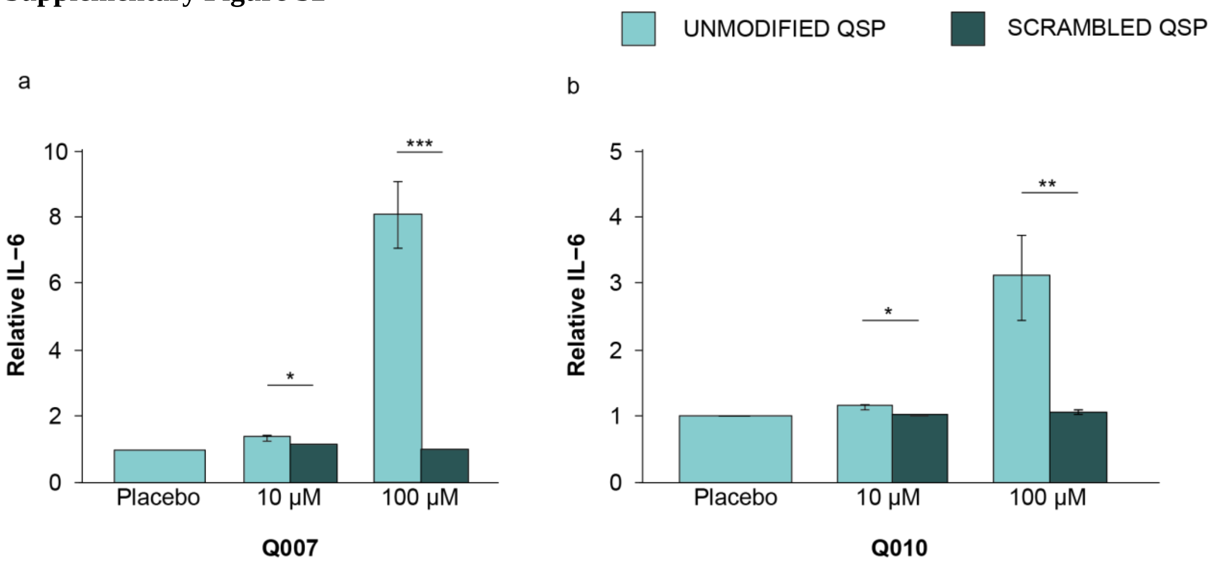

Supplementary Figure S2. Results of IL-6 ELISA after 24h incubation of splenocytes with different concentrations QSP in its unmodified (red) or scrambled (green) form. a) Q007; b) Q010. Bars represent means, with error bars representing s.e.m. Asterisks indicate the following criteria of statistical significance: *p≤0.05 **p≤0.01 ***p≤0.001 (Two-sided t-test between unmodified and scrambled form at specific concentration).

Supplementary Figure S3

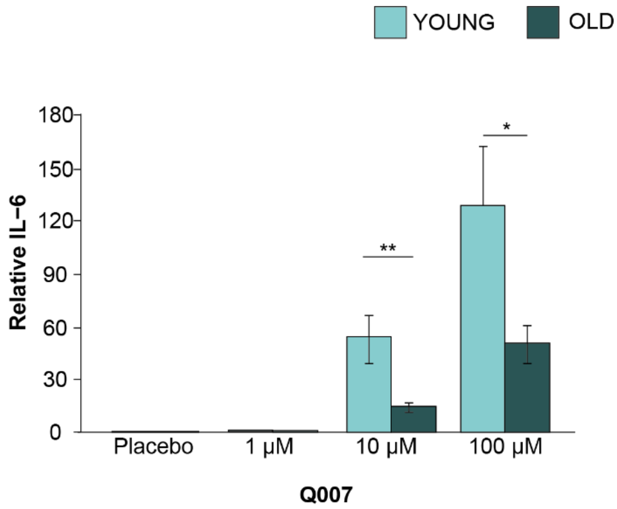

Supplementary Figure S3. Age-dependent results of IL-6 ELISA after 24h incubation of human PBMC’s with different concentrations Q007. Bars represent means, with error bars representing s.e.m. Asterisks indicate the following criteria of statistical significance: *p≤0.05 **p≤0.01 (Two-sided t-test between different ages within the same QSP concentration).

Supplementary Figure S4

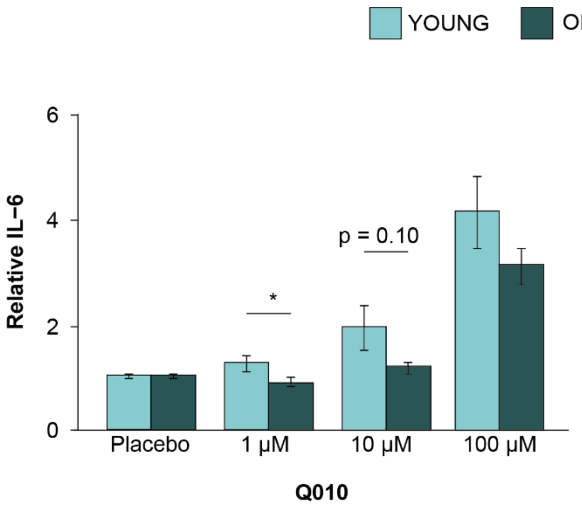

Supplementary Figure S4. Age-dependent results of IL-6 ELISA after 24h incubation of human PBMC's with different concentrations Q010. Bars and numbers on top represent means, with error bars representing s.e.m. Asterisks indicate the following criteria of statistical significance: *$p \leq 0.05$ (Two-sided t-test between different ages within the same QSP concentration).

Supplementary Table S1

Table S1. Average recovery, matrix effect and process efficiency of Q010 in human plasma determined on Xevo.

QSP	Parameter	Matrix 1	Matrix 2	Matrix 3	Overall	SD	RSD
**Q010**	**Process yield**	**89.6%**					
	**Recovery**	107.2%	107.0%	107.9%	**107.4%**	0.4%	0.4%
	**Matrix Effect**	80.8%	78.8%	80.5%	**80.0%**	1.1%	1.3%
	**Process efficiency**	86.6%	84.4%	86.8%	**86.0%**	1.4%	1.6%

Supplementary Table S2

Table S2. Summary of coefficients (Coeff) for plasma cytokine concentration ($PapR_7I$ positive vs. negative) using propensity score overlap weighted linear regression (n = 39).

Cytokine	Coeff (95% CI)<br>$PapR_7I$ positive vs. negative	P-value
IL-6	1.38 (-4.28; 7.05)	0.635
IFNγ	-2.21 (-3.95; -0.46)	**0.018**
MCP-1	6.81 (-32.9; 46.5)	0.739
IL-10	-0.73 (-3.10; 1.63)	0.548
IL-18	-71.8 (-159; 15.0)	0.114
IL-23	-0.51 (-4.65; 3.62)	0.809

IL-1β	-6.01 (-21.1; 9.07)	0.440
IL-33	-11.5 (-26.0; 3.02)	0.129
